# Overnutrition and Associated Factors: A Comparative Cross-Sectional Study between Government and Private Primary School Students in Gondar Town, Northwest Ethiopia

**DOI:** 10.1155/2020/3670895

**Published:** 2020-10-01

**Authors:** Mohammed Sied Ali, Chanyalew Worku Kassahun, Chalachew Adugna Wubneh

**Affiliations:** ^1^Department of Pediatrics and Child Health Nursing, School of Nursing, College of Medicine and Health Sciences, University of Gondar, Gondar, Ethiopia; ^2^Department of Comprehensive Nursing, College of Medicine and Health Sciences, University of Gondar, Gondar, Ethiopia

## Abstract

**Introduction:**

Childhood overnutrition is a public health problem in low- and middle-income countries because its effect is likely to progress into adulthood that results in developing noncommunicable diseases at a younger age. There is no such previous comparative study that investigated this issue. Therefore, the aim of this study is to assess the prevalence and associated factors of overnutrition among government and private primary school students in Gondar town, northwest Ethiopia.

**Methods:**

Institution-based comparative cross-sectional study was conducted from March 5 to April 9, 2019. A multistage sampling technique was used to select 736 participants. Data were collected through face-to-face interview. Data was entered into EPI-info version 7.2.1.0 and exported to SPSS version 20 for analysis. Bivariable and multivariable logistic regressions were carried out to identify associated factors with overnutrition. Statistical significance was declared using *p* value< 0.05.

**Results:**

The overall prevalence of overnutrition was 9.1%. The prevalence was higher among private schools (51 (14%)) than government (16 (4.3%)). Eating habit while watching television (AOR = 4.08, 95%CI: 1.10–15.16) and not having close friend (AOR = 3.72, 95%: CI: 1.21–2 11.48) were significantly associated with overnutrition in the government schools, while no father education (AOR = 2.59, 95%:CI:1.05–6.39), sweet food preference (AOR = 2.86, 95%:1.19–6.87), fat consumption more than three days per week (AOR = 3.79, 95%CI:1.61–8.91), eating habit while reading (AOR = 4.95, 95%CI:2.29–10.70), and vigorous-intensity sports (AOR = 2.23, 95%:1.02–4.86) were associated with overnutrition in private schools.

**Conclusion:**

Prevalence of overnutrition was higher among private than government schools. Hence, it requires attention through creating awareness about healthy diet, healthy lifestyle, and physical activity in collaboration with health and education sectors.

## 1. Introduction

Overnutrition is defined as abnormal or excessive accumulation of fat in the adipose tissue that may affect the health status of individuals. It is the sum of overweight and obesity [1]. World Health Organization (WHO) defines overweight as body mass index (BMI) for age, Z-score between +1 and + 2 standard deviation (SD), and obesity as BMI for age and Z-score > +2 SD [2]. The prevalence of overnutrition among children and adolescents (aged 5–19) has risen dramatically from 4% in 1975 to just over 18%; over 340 million children and adolescents aged 5–19 were overweight or obese worldwide in 2016 [3]. Overweight and obese children are more likely to stay obese into adulthood and more likely to develop chronic diseases like diabetes mellitus, hypertension and coronary artery disease at a younger age, and increased future risks of breathing difficulties, fractures, insulin resistance, cancer, asthma, and psychological comorbid effects [4]. In addition, overnutrition is related to more deaths worldwide than undernutrition. Over 71% of all deaths globally are due to noncommunicable diseases (NCD) [4].

Lifestyle and diets of children from urban areas make it sufficiently more risky to be overweight compared to children living in deprived rural environments [5]. Another challenging issue related to overnutrition is poor school performance of the students in their academic score [6]. Many studies emphasized the positive relationship between obesity and poor school performance [7]. Childhood obesity can profoundly affect children's physical, social, and emotional well-being. It is also associated with a lower quality of life experienced by the child [8]. A study in Africa and other developing countries has documented an emerging trend of malnutrition with overweight and obesity increasing at an alarming rate in comparison to undernutrition [9].

This shifting comes with increased access to high-calorie foods and less strenuous jobs resulting in many individuals having a positive energy balance and hence becoming overweight or obese [10]. In Sub-Saharan Africa, most nutrition efforts have focused on undernutrition among children, but the focus is low on overnutrition [11]. Globalization, improving economic conditions, and changing dietary habits in developing countries are reported as responsible for the rapid increase in overnutrition [12].

Factors contributing to overnutrition are foods that are high in fats, sugars, energy-dense foods, and increasing low-intensity activity due to new modes of transportation and rapid urbanization, which continue to dramatic changes in living environments as well as in diets and lifestyles that promote positive energy balance [13]. The coexistence of undernutrition and overnutrition also known as double burden of malnutrition (DBM) is a recent emerging nutritional problem stated in low- and middle-income countries like Ethiopia [14].

However, the federal ministry of health and other organizations like Ethiopian public health institute and Ethiopian nutritional institute tried to disseminate the information in different media and health institutions about healthy eating habit and regular physical activity to reduce the burden of nutritional problems, but the problem is shifting from undernutrition to overnutrition. Even if there were some studies about overnutrition among children in Ethiopia, but there is limited studies in epically comparing burden of overnutrition among private and governmental school children with significant economical different population in the same seating.

Therefore, this study was conducted to determine the prevalence and associated factors of overnutrition among government and private primary school children in Gondar town. The finding of this study will be important to take the appropriate measurement for the problem of the targeted population.

## 2. Methods

### 2.1. Study Design and Period

Institution-based comparative cross-sectional study design was conducted from March 5/2019 to April 9/2019.

### 2.2. Study Area and Population

The study was conducted among the second cycle (Grades 5–8) primary school students in Gondar town. The town is found 748 km far away from Addis Ababa. Gondar is the fourth largest city in Ethiopia with a population of 358,257 people according to Gondar town statistics office. Gondar is the city in the central Gondar zone which is in the Amhara region located in the Northwest part of Ethiopia.

### 2.3. Source Population

It comprises all second cycle (5–8 grades) primary school students who were registered in 2018/2019 academic year in Gondar town.

### 2.4. Study Population

It comprises all second cycle (5–8 grades) primary school students who were selected randomly.

### 2.5. Inclusion Criteria

They comprise all primary school students, whose grades were 5–8 and those students who are under the age of 18 years.

### 2.6. Exclusion Criteria

They comprise students who were critically sick during data collection and those who were unable to communicate verbally.

### 2.7. Sample Size Determination

The sample size for this study was estimated by two methods for the two specific objectives; lastly, the larger sample was taken, the first sample size was determined by using a double population proportion formula by considering the following statistical assumptions: 95% CI, taking the prevalence of overnutrition, *p*1 = 23.0% in private and *p*2 = 4.3% in government from the previous study done in Addis Ababa which was conducted in both private and government schools (25):

(1) $$\begin{array}{c} n=\frac{{\left(Z\alpha 2|+|Z|\beta \right)}^{2}\left(p1\left(1-p1\right)+p2\left(1-p2\right)\right)}{{\left(p1-p2\right)}^{2}}, \end{array}$$

where *p*1 is proportion of overnutrition in private, *p*2 is proportion of overnutrition in the government school,  power = 0.84, confidence level = 1.96, 10% nonresponse rate added, and design effect 2, and the calculated sample size is equal to 108 for each and total sample size  is 216.

For the factors, the sample size was calculated using EPI-info from the factors that are significantly associated with overnutrition in the previous studies to select the factor having a maximum possible sample size by using 95% confidence interval and power 80% for all variables.

So, the total sample size for this study is 774. Since the ratio is one to one, 387 from private and 387 from government schools were selected and participated in the study.

### 2.8. Sampling Procedure

Multistage sampling method was used to select the study participants. All primary schools (31 primary schools) in Gondar town were stratified into government and private schools. Lottery method was used to select 40% of the 31 schools based on WHO tools for assessment guideline [15]. Seven schools from 17 government schools and 5 private schools from 14 private schools were selected randomly. From each of the 12 schools, all sections of Grades 5, 6, 7, and 8 were separately enlisted and one section from each grade was selected by lottery method. A total of 7,261 children were enumerated from the recruited schools. Finally, to select 774 study participants, systematic random sampling technique was used from the selected sections. Proportional allocation used the formula ni = (*n*/*N*) × Ni, where *n* is total sample size to be selected, *N* is total population, Ni is total population of each school, and ni is sample size from each school.

### 2.9. Data Collection Tools and Procedures

Structured, pretested, and interviewer-administered questionnaire was used to collect the data. Most of the questions were adapted from previous study [16] and physical activity related questions were adopted from the Global Physical Activity Questionnaire (GPAQ) analysis guide [17]. Dietary related questions were adopted from the Food and Nutrition Technical Assistance [18].

The selected children were well informed about the procedures. For those well-informed children, a few and too simple questions were sent to home that would be completed with their parents (any guardian) and were returned the following day. All the participating students were interviewed at school outside the classroom to keep responding freely and correctly. Anthropometric measurement of weight and height was done using calibrated measuring tools. Height was measured to the nearest 0.5 cm in standing position at Frankfurt plane with the occipital, shoulder, and the buttock touching the vertical stand using a stadiometer. Weight was measured to the nearest 0.5 kg using weighing scale while wearing light clothes and shoes. Data were collected by four BSc nurses and data collectors were supervised by two MSc nurses.

### 2.10. Data Quality Control

The questionnaire was prepared first in English and then it was translated to the Amharic language and back to English language. It was reviewed by language experts for consistency of translation of the language and it was reviewed by nutritionists to check its appropriateness for assessing overnutrition in children. Data collectors and supervisors were trained for two days about the whole procedures of data collection.

The data were collected after pretest was conducted on 5% of primary school students other than those included in the actual study; then ambiguous questions were corrected and unnecessary questions were excluded based on the pretest.

The investigators and supervisors had day-to-day on-site supervision during the whole period of data collection. Weighting scale was calibrated and placed in level surface before and after each measurement. Continuous checkup of scales was carried out throughout the data collection. The completeness of the questionnaire was checked before data entry.

### 2.11. Data Processing and Analysis

Data were first coded and entered using EPI-info version 7.2.1.0 for data exploration and cleaning. The cleaned data were exported to SPSS version 20 statistical packages for statistical analysis. Descriptive and summary statistic was carried out to describe study participants according to different characteristics. Independent variables for sociodemographic characteristics such as age, monthly family income, and family size were analyzed after conversion into categorical variables.

Magnitude of overnutrition was determined by importing age, sex, height, and weight of the participants into WHO Anthro-Plus version 1.0.4; then BAZ was imported to SPSS. BAZ status was recoded to overnutrition and non-overnutrition. The binary logistic regression model was fitted to identify factors associated with overnutrition. Three models were fitted independently, the whole sample (for government, private, and combined), for the government school and for private schools. The model fitness for each model was tested by Hosmer–Lemeshow goodness of fitness, and the results of the test showed that the models were fit for each three models.

To check an interaction or effect modification of the independent variables, multicolinearity of the independent variables was checked using the variance inflation factor. There is no multicolinearity among the independent variables. Bivariable associations between dependent and several independent variables were examined one by one and those variables with *p* value less than 0.2 were entered to multivariable logistic regression. Multivariable logistic regression analysis was employed to identify factors associated with overnutrition by controlling the effect of potential confounding variables. Odds ratio (OR) with 95% CI was computed to assess the level of association and statistical significance. Statistical significance was declared using *p* value less than 0.05. The result of this study is described in texts, tables, and graphs.

### 2.12. Ethical Considerations

Ethical clearance was obtained from the institutional review board of University of Gondar. Written informed consent was obtained from a parent or guardian for participants under 16 years old, after informing them all the purpose, benefit, risk, the confidentiality of the information, and the voluntary nature of the participation in the study. Then assent was obtained from the children. Participation was on voluntary basis and confidentiality was maintained to encourage accurate and honest self-disclosure. In addition to ethical clearance support, the latter was communicated with all concerned offices.

## 3. Results

### 3.1. Sociodemographic Characteristics of the Parents

A total of 736 children-parent pairs (50.5% from government and 49.5% from private schools) participated in the study with 95.09% response rate. The median monthly income of the family was 4000 with Inter-Quartile Range (IQR), 2000–6000 Ethiopian birr. One-third (33.6%) of children in government schools and approximately half (48.1%) of the children's father in private schools attended college and above level of education (Table 1).

### 3.2. Children's Sociodemographic Characteristics

Among a total of 736 children who participated in the study, almost half (51.1%) were males and 48.9% were females in government schools. In private schools, 53.3% were females and 46.7% were males. The median age of the children was 13 years with IQR, 13–15 years. Two hundred seven (55.6%) from government and one hundred fifty-eight (43.4%) from private schools were above 14 years old (Table 2).

### 3.3. Dietary Habit and Food Preference-Related Characteristics

Among the total participants, the majority (87.9%) in government schools and 91.5% in private schools of the children had breakfast before going to school. Two hundred twenty-five (60.5%) of the children from government and two hundred eighteen (59.9%) from private schools prefer sweet foods (Table 3).

### 3.4. Physical Activities and Sedentary Lifestyle-Related Characteristics

Among a total of 736 children, 60.5% in government and 62.4% in private schools were involved in vigorous-intensity sports outside their school for at least 10 minutes, while for moderate-intensity sports 62.9% from government and 64.0% from private schools were involved outside their school for at least 10 minutes. Two hundred ninety-eight (80.1%) of the children in government and majority (86.0%) of the children from private schools traveled on foot/rode bicycle for at least 10 minutes (Table 4).

### 3.5. The Overall Magnitude of Overnutrition among Primary School Students in Gondar Town

**Figure 1:**
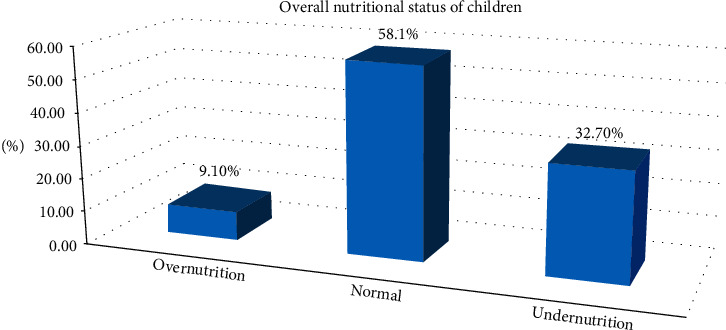
The nutritional status of primary school students in Gondar town, 2019.

The overall magnitude of overnutrition among children in primary schools of Gondar town was 9.1% with 95% CI (7.1–11.1), of which overweight accounted for 7.6% and obesity accounted for the rest, 1.5%. It was higher in female, 13% (10.6% overweight and 2.4% obese), than male, 5.1% (4.5% overweight and 0.6% obese), children. The comparison of magnitude of overnutrition among governmental and private school was calculated separately. The magnitude of overnutrition was higher among private school children, 14% (11.8% overweight and 2.2% obese), than government school children, 4.3% (3.5% overweight and 0.8% obese) (Figure 1).

Comparison of magnitude of overnutrition among government and private primary school students.

**Figure 2:**
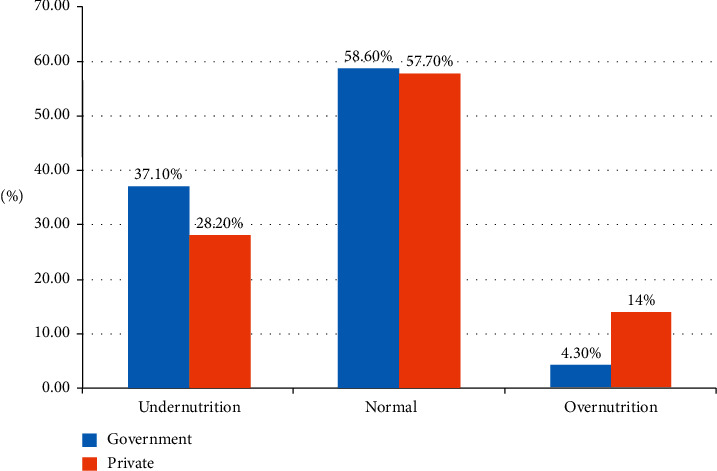
The comparison of nutritional status of primary school students in Gondar town, 2019.

The magnitude of overnutrition was higher in the private school students, 14%, with 95% CI (10.3–17.6) than the government school, 4.3%, with 95% CI (2.3–6.5). This difference was statistically significant (*p* value <0.001). The percentage of underweight among government school students was higher (37.1%) as compared to the students of private schools (28.2%) (Figure 2).

### 3.6. Factors Associated with Overnutrition among Primary School Students

#### 3.6.1. Factors Associated with Overnutrition among Government Schools

Bivariate analysis was carried out and six variables were associated with overnutrition among governmental schools. In multivariable analysis, two of them were found to be significantly associated. Children who had no close friend in the school or community were 3.7 times more likely to be overnourished as compared to those who had close friend (AOR = 3.72, 95% CI: 1.21–11.47), and children who had a habit of eating while watching television were four times more likely to be overnourished as compared to those who had no a habit of eating while watching television (AOR = 4.08, 95% CI: 1.10–15.16).

#### 3.6.2. Factors Associated with Overnutrition among Private School Students

In the bivariate analysis, the factors associated with overnutrition among private primary school students, children who had father with no formal education were nearly 2.6 times more likely to be overnourished as compared to higher education (AOR = 2.59, 95%, CI:1.05–6.39); children who prefer sweet foods were 2.8 times more likely to develop overnutrition as compared to those who did not prefer sweet foods (AOR = 2.86, 95% CI:1.19–6.87); children who had a habit of eating while reading were nearly 5 times more likely to be overnourished as compared to those who did not have a habit of eating while reading (AOR = 4.95, 95% CI: 2.23–10.70); those who consumed fatty foods more than three days per week were 3.9 times more likely to develop overnutrition as compared to those who consumed fatty foods less than three times per week (AOR = 3.79, 95% CI: 1.61–8.91) and those not involved in vigorous-intensity sports were 2.4 times more likely to be over nourished (AOR = 2.23, 95% CI:1.02–4.86).

### 3.7. Overall Factors Associated with Overnutrition among Primary School Students

Children who attended their education in private schools were 3.3 times more likely to develop overnutrition as compared to government schools (AOR = 3.33, 95% CI: 1.58–7.03).

Children who prefer sweetened foods were 2.6 times more likely to be overnourished as compared to those who did not prefer sweetened foods (AOR = 2.66, 95% CI:1.24–5.72). Eating habit while reading was significantly associated with overnutrition (AOR = 3.52, 95% CI: 1.85–6.70), 3.5 times more likely to develop overnutrition as compared to those who did not have a habit of eating while reading. Children who consumed fatty foods more than three days per week were 3.5 times more likely to be overnourished as compared to those who did not consume fatty foods (AOR = 3.5, 95% CI: 1.60–7.65); those who consumed vegetables less than three days per week were 2.5 times more likely to develop overnutrition (AOR = 2.51, 95%CI: 1.23–5.04) and children who were not involved in vigorous-intensity sports were 2.7 times more likely to be overnourished as compared to those involved in vigorous-intensity sports (AOR = 2.7, 1.33–5.49) (Table 5).

## 4. Discussion

This study revealed the prevalence and associated factors of overnutrition among government and private primary school students in Gondar town. Accordingly, the overall prevalence of overnutrition was 9.1% (7.1–11.1). The school type specific prevalence of overnutrition was 14% among private and 4.3% among government schools which shows there was a significant difference in prevalence of overnutrition among private schools and government schools (*p*=0.001). The possible explanation for this variation may be that those students attending private school are from families having good economic status compared to government school students. For this reason, private school students may consume foods that expose to obesity and overweight.

The overall prevalence of overnutrition in this study was comparable with the findings from studies conducted in Addis Ababa, Bole subcity; the overall prevalence of overnutrition among private and government schools was 9.8% [19] and in Lome Togo 7.1% [20] for both government and private. The overall prevalence of overnutrition in this study was slightly lower than studies conducted in Ethiopia: Addis Ababa 12.7% [21], Dire Dawa 20.5% [16], and Jimma town 13.3% [22]. This regional discrepancy might be due to environmental variation among the study cities and might be more urbanized than the study area. It also might be due to variation in eating habit and lifestyle across the population among Ethiopian. In Ethiopia, there are different cultural and religious practices which affect food preference and feeding habits; this directly affects the nutritional status of the children [23].

The overall prevalence in this study was also lower than studies conducted in Kenya, 19.0% (11); Tanzania, 22.6% (23) and another study in Tanzania, 20.0% [24]; Egypt, 31.2% [25]; Nigeria, 17.4% [9]; China, 17.5% [26] and 20% [27]; Ukraine, 17.6% [28]; Iran, 18.1% [28]; and Lithuania, 16.0% [29]. The difference might be due to the socioeconomic, cultural, and lifestyle difference between those countries. This variation could also be due to difference in culture of physical activities since there is habit of using on-foot travel in the study area and less exposure to sedentary lifestyle than other nations due to less access to vehicle as compared to other countries.

Children in private primary school had a higher proportion of overnutrition (14%) than that of the children of government primary schools (4.3%). This finding is supported by the findings of the studies conducted in different areas of the world: Addis Ababa 16.0% private and 4.3% government [21], Dire Dawa 45.6% private and 11.0% government [16], Bole subcity 8.1% private and government 1.7% [19], Kenya 23.6% private and 7.3% government [30], and in another study in Kenya 29.0% private and 11.5% government [11]; in Tanzania, the prevalence was higher among private than government with *p*=0.021 [31], Egypt 57% private and 42.3% but much higher than this study, Iran [28], Mysore city, India14.9% private and 0.2% government [32], and in another study India 27.9% among private and only 2.45% among government [33]. This discrepancy might be because parents with higher socioeconomic background of private school children would expose them for higher adoption of unhealthy dietary habits (energy-dense foods, sweetened foods, and fatty animal products) and sedentary lifestyle due to transportation to and from school by vehicle than government school children.

The associated factors of overnutrition among government schools were eating habit of children while watching TV. This finding is in line with the study conducted in Addis Ababa, Bole subcity [19]. Another study conducted in India [34] showed that overnutrition was much higher among those who had a habit of watching TV for more than three hours per day and studies in eight European countries stated that eating habit while watching TV was a significant factor for overnutrition. It was found that the negative relationship between never watching TV at meal and overnutrition still remain significant [35]. Watching TV while eating might allow less attention to meal and result in eating more for longer duration of time which causes overfeeding. Therefore, the body may get extra calories more than the body requires to be properly utilized which are excess for specially already sedentary lifestyle [36].

Children who had no close friend in the school or community among government school students were associated with overnutrition. This finding was consistent with a study conducted in Dire Dawa [16]. This might be because children who had no close friend are exposed to sedentary lifestyle; usually, they do not play outdoor games or physical exercise alone but rather they stay at home and consume more calories. In addition, they may be addicted to electronic game and watching TV which further exposed to overweight and obesity.

According to this finding, low parental education among private schools was significantly associated with overnutrition. This finding was in agreement with other studies conducted in Jimma [22] and Lithuania [29], but in Pakistan [37], it was higher level of education that was associated with overnutrition. This might be because parents with lower educational status may not have adequate knowledge regarding nutrition, food diversity, feeding condition, and exercise because these are the important characteristics the parents should have for better nutritional management of their children and good health outcome.

Children in private schools and overall sample, who preferred sweetened foods, were more likely to be overnourished as compared to those who did not prefer sweetened foods. This finding was similar to other different studies, Addis Ababa [21], Dire Dawa [16], Saudi Arabia [38], and India [39] which revealed that sweetened foods preference was found to be significantly associated with overnutrition. This is because private schools children might have less restriction on food and snack choices compared with those in government schools [40]. This might also be because sweetened food items are energy-dense foods which result in positive energy balance to their consumers and sweet foods contain high sugar content which results in weight gain and becoming overweight [41].

The current study found a significant association between reduced vegetable (consumption less than three days per week), but not fruit, intake. Vegetable consumption was significantly associated with overnutrition among the overall (both private and government) in children. This result was similar to a study conducted in Jimma [22] and Saudi Arabia [38]. However, longitudinal studies among overweight children and adults showed marked associations between increased consumption of fruits and vegetables and slower weight gain, but only one-half of the children longitudinal studies indicated significant inverse associations between fruit and vegetable intake and overnutrition [42]. There is a fact that vegetable consumption can be one aspect of weight balancing and reduction of overweight [42].

Fat consumption more than three days per week was significantly associated with overnutrition among private school and overall in this study. This finding is consistent with studies conducted in Addis Ababa [21], Bole subcity [19], and Romania [43]. According to another study conducted in Switzerland [44], this might be because dietary fat induces overconsumption and weight gain through its low satiety properties and high caloric density which leads to overnutrition [45]. This might also be due to causal relation between dietary fat and body fat, consumption of more fatty foods lead to surplus energy storage in the adipose tissue [46].

In his study, overnutrition was higher among private school and overall children who had a habit eating while reading than those who did not have this habit. This finding was supported by other studies [36]. This may be due to the fact that those students attending school in private come from high-income family. Related with high income, those students may eat high-calorie food and may have sedentary lifestyle.

This study found that children in private and overall sample who did not engage in physical exercise were significantly associated with overnutrition. The finding that overnutrition was associated with lower levels of physical activity highlights the important role that physical activity, particularly vigorous activity, plays in preventing childhood obesity. The present findings were consistent with the growing evidence showing that physical inactivity is a leading factor in obesity during childhood and adolescence [47]. This finding was highly supported by several studies conducted in different areas, Addis Ababa [21], Dire Dawa [16], Jimma [22], Lagos Nigeria [48], Saudi Arabia [48], India [4], India [39], Romania [43], and Central Java [5], which revealed that physical inactivity (sedentary lifestyle) has been a well-documented cause of overnutrition in children.

In private schools, the low level of physical activity could be due to several reasons. Most of the children in private are either driven in car to school by their parents or use the school service (bus). This might expose them to energy storage without expenditure, in contrast to the government school most of the children travel to school on foot [49].

## 5. Conclusion

The overall prevalence of overnutrition in Gondar town was 9.1%. The prevalence of overnutrition was significantly higher among children in private schools (14%) than government schools (4.3%). Among the factors, eating habit while watching television and not having close friend are important factors contributing to overnutrition among children in government schools. No formal father education, sweet food preference, fat consumption more than three days per week, eating habit while reading, and not engaging in vigorous-intensity sports are important factors contributing to the high prevalence among private school children.

## Figures and Tables

**Table 1:** Parents sociodemographic characteristics among primary school students in Gondar town, 2019 (*n* = 736).

Variables	Response	Government, (*n* = 372)		Private, (*n* = 364)	
		Frequency	%	Frequency	%
Paternal education	Unable to read and write	28	7.5	11	3.0
	Able to read and write	134	36.0	66	18.1
	Primary school level	34	9.1	42	11.5
	Secondary school level	51	13.7	70	19.2
	College and above	125	33.6	175	48.1
Maternal education	Unable to read and write	53	14.3	21	5.8
	Able to read and write	110	29.6	63	17.3
	Primary school level	58	15.6	42	11.5
	Secondary school level	60	16.1	86	23.6
	College and above	91	24.3	152	42.8
Occupation of the head of household	Merchant	117	31.5	118	32.4
	Private employee	84	22.6	83	22.8
	Government employee	100	26.9	118	32.4
	NGO	23	6.2	23	6.3
	Daily laborer	31	8.6	7	1.9
	Pension	8	2.2	7	2.0
	Farmer	4	1.1	3	1.0
	Housewife	5	1.3	4	1.1
Religion	Orthodox	273	73.4	280	76.9
	Muslim	84	22.6	51	14.0
	Protestant	15	4.0	33	9.1
Monthly income	Below the median	230	61.8	134	36.8
	Above the median	142	38.2	230	63.2
Family size	<5	100	26.9	208	57.1
	≥5	272	73.1	156	42.9
Family car owner	Yes	26	7.0	45	12.4
	No	346	93.0	319	87.6

**Table 2:** Children's sociodemographic characteristics among primary school students in Gondar town, 2019 (*n* = 736).

Variables	Response	Government, *n* = 372		Private, *n* = 364	
		Frequency	%	Frequency	%
Sex	Male	190	51.1	170	46.7
	Female	182	48.9	194	53.3

Age	11–13 years	165	44.4	206	56.6
	14–17 years	207	55.6	158	43.4

Grade	Grade 5	71	19.1	78	21.4
	Grade 6	107	28.8	108	29.7
	Grade 7	90	24.2	119	32.7
	Grade 8	104	28.0	59	16.2

Having close friends	Yes	292	78.5	294	80.8
	No	80	21.5	70	19.2

**Table 3:** Dietary habit and food preference related characteristics among primary school students in Gondar town, 2019 (*n* = 736).

Variables	Response	Government, *n* = 372		Private, *n* = 364	
		Frequency	%	Frequency	%
Breakfast intake	Yes	327	87.9	333	91.5
	No	45	12.1	31	8.5
Number of days breakfast intake per week	Once	14	4.2	12	3.6
	2–4 days	98	29.7	115	34.3
	5 and above days	218	66.1	208	62.1
Snack intake	Yes	320	86.0	319	87.6
	No	52	14.0	45	12.4
Snack intake per day	Once	276	86.3	284	78.0
	≥2 times	44	7.8	38	10.4
Meal frequency per day	1–2 times	70	18.9	53	14.6
	3 times	234	62.9	241	66.4
	≥4 times	68	18.2	69	19.0
Place of getting lunch	Home	351	94.4	337	92.6
	Outside home	10	2.7	16	4.4
	Did not use lunch	11	3.0	11	3.0
Sweet food preference	Yes	225	60.5	218	59.9
	No	147	39.5	146	40.1
Foods bought other than regular meal	Not buy	107	28.8	88	24.2
	Biscuit/chocolate	127	34.2	148	40.7
	Cake/ice cream	94	25.3	92	25.2
	Chips	26	7.0	20	5.5
	Potato	5	1.3	5	1.4
	Cabbage	5	1.3	7	2.2
	Bread	8	2.2	4	1.1
Eating habit while watching TV	Yes	202	54.3	222	61.0
	No	170	45.7	142	39.0
Eating habit while reading	Yes	59	15.9	73	20.1
	No	313	84.1	291	79.9
Meat/poultry/fish	No	150	40.3	92	25.2
	1-2 times per week	1 57	42.2	158	43.4
	≥3 times per week	65	17.5	114	31.3
Fat/oil consumption	No	167	44.9	158	43.5
	1-2 times per week	109	29.3	105	28.8
	≥3 times per week	96	25.8	101	27.7
Fruit consumption per week	No	44	11.8	27	7.4
	1 day	134	36.0	143	39.3
	≥2 days	193	51.9	194	53.3
Vegetable consumption per week	No	37	9.9	30	8.2
	1-2 days	185	49.7	190	52.2
	≥3 days	150	40.3	144	39.6
Soft drink consumption per week	No	55	14.8	80	22.0
	1-2 days	205	55.1	186	51.1
	≥3 days	112	30.1	98	26.9

**Table 4:** Physical activities and sedentary lifestyle-related characteristics among primary school students in Gondar town, 2019 (*n* = 736).

Variables	Response	Government (*n* = 372)		Private (*n* = 364)	
		Frequency	%	Frequency	%
Walk on foot/ride bicycle for at least 10 min	Yes	298	80.1	313	86.0
	No	74	19.9	51	14.0
Number of days to walk/ride bicycle per week	1-2 days	38	12.8	84	26.6
	3-4 days	57	19.1	74	23.4
	≥5 days	203	68.1	158	50.0
Vigorous-intensity sports for at least 10 min	Yes	225	60.5	227	62.4
	No	147	39.5	137	37.6
Number of days for vigorous sports per week	1-2 days	144	63.7	150	65.5
	3-4 days	71	31.4	64	27.9
	≥5 days	11	4.9	15	6.6
Time spend for vigorous sports per day	<30 min	99	44.6	102	44.7
	30–60 min	79	35.6	89	39.0
	≥60 min	44	19.8	37	16.2
Moderate-intensity sports for at least 10 min	Yes	234	62.9	233	64.0
	No	138	37.1	131	36.0
Number of days for moderate physical activities/sports per week	1-2 days	139	59.6	164	70.1
	3-4 days	71	30.5	53	22.6
	≥5 days	23	9.9	17	7.3
Time spend for moderate physical activities/sports per day	<30 min	73	31.3	74	31.6
	30–60 min	103	44.2	122	52.1
	≥60 min	57	24.5	68	16.3
Mode of transportation to and from school	On foot	309	83.1	163	44.8
	By vehicle	63	16.9	201	55.2
Spend free time	Watching TV/video	147	39.5	158	43.4
	Play mobile game	59	15.9	51	14.0
	Extra home activity	154	41.4	148	40.7
	Reading fiction	3	0.8	2	0.5
	Playing with friends	5	1.3	2	0.5
	Going to religious area	4	1.1	3	0.9
Time spent by watching TV/video	<2 hrs	168	46.0	159	44.5
	2–3 hrs	136	37.3	136	38.1
	≥3 hrs	61	16.7	62	17.4
Time spend sitting/reading per day	<3 hrs	206	55.4	228	62.6
	3–5 hrs	135	36.3	115	31.6
	≥5 hours	31	8.3	21	5.8

**Table 5:** Multivariable logistic regression of factors associated with overnutrition among governmental primary school students, 2019 (*n* = 736).

Variables	Response	Overnutrition		COR (95% CI)	AOR (95% CI)	*p* value
		No	Yes			
Factors associated with overnutrition among governmental primary school students						
Sex	Male	185	2	1.0	1.0	0.331
	Female	171	14	3.24 (1.02–10.25)	1.85 (0.53–6.46)	
Close friend	Yes	287	5	1.0	1.0	0.022^*∗*^
	No	69	11	5.34 (1.92–14.86)	3.72 (1.21–11.47) ^*∗*^	
Fat intake per week	No	163	4	1.0	1.0	
	1-2 days	105	4	1.55 (0.38–6.34)	1.97 (0.45–8.47)	0.362
	≥3 days	88	8	3.70 ^(^1.08–12.64)	2.93 (0.80–10.72)	0.103
Eating while watching TV	Yes	190	13	3.78 (1.06–13.51)	4.08 (1.10–15.16) ^*∗*^	0.035^*∗*^
	No	166	3	1.0	1.0	
Vigorous sports	Yes	220	5	1.0	1.0	0.253
	No	136	11	3.56 (1.21–10.46)	2.26 (0.56–9.24)	
Moderate sports	Yes	230	6	1.0	1.0	0.576
	No	126	10	3.04 (1.08–8.56)	1.47 (0.37–5.80)	

Factors associated with overnutrition among private primary school students						
Sex	Male	155	16	1.0	1.0	0.737
	Female	158	35	2.14 (1.14–4.03)	0.87 (0.39–1.93)	
Father education	No formal	59	17	2.13 (1.05–4.31)	2.59 (1.05–6.39) ^*∗*^	0.038^*∗*^
	Primary	39	3	0.56 (0.16–2.00)	0.76 (0.16–3.55)	0.732
	Secondary	60	10	1.23 (0.56–2.76)	1.93 (0.73–5.05)	0.181
	Above college	155	21	1.0	1.0	
Breakfast intake	Yes	291	42	1.0	1.0	0.264
	No	22	9	2.83 (1.22–6.56)	1.90 (0.61–5.87)	
Sweet food preference	Yes	175	43	4.23 (1.93–9.31)	2.86 (1.19–6.87) ^*∗*^	0.018^*∗*^
	No	138	8	1.0	1.0	
Eating while reading	Yes	44	29	8.05 (4.25–15.27)	4.95 (2.29–10.70) ^*∗*^	0.001^*∗*^
	No	269	22	1.0	1.0	
Fat intake per week	No	147	11	1.0	1.0	
	1-2 days	96	9	1.3 (0.50–3.13)	1.34 (0.48–3.71)	0.573
	≥3 days	70	31	5.91 (2.81–12.45)	3.79 (1.61–8.91) ^*∗*^	0.002^*∗*^
Walk/ride bicycle	Yes	278	35	1.0	1.0	0.441
	No	35	16	2.63 (1.82–7.22)	1.46 (0.55–3.85)	
Vigorous sports	Yes	211	16	1.0	1.0	0.042^*∗*^
	No	102	35	4.52 (2.39–8.55)	2.23 (1.02–4.86) ^*∗*^	
Moderate sports	Yes	214	19	1.0	1.0	0.077
	No	99	32	3.64 (1.96–6.73)	2.03 (0.92–4.48)	

Factors associated with overnutrition among primary school students						
School type	Government	356	16	1.0	1.0	0.002^*∗*^
	Private	313	51	3.62 (2.02–6.48)	3.33 (1.58–7.03) ^*∗*^	
Sex	Male	340	20	1.0	1.0	0.302
	Female	329	47	2.43 (1.41–4.18)	1.41 (0.73–2.73)	
Close friend	Yes	542	44	1.0	1.0	0.206
	No	127	23	2.23 (1.30–3.82)	1.55 (0.78–3.10)	
Sweet food preference	Yes	387	56	3.71 (1.91–7.21)	2.66 (1.24–5.72) ^*∗*^	0.012^*∗*^
	No	282	11	1.0	1.0	
Eating while watching TV	Yes	375	50	2.30 (1.30–4.08)	1.82 (0.89–3.74)	0.10
	No	294	17	1.0	1.0	
Eating while reading	Yes	98	34	6.0 (3.55–10.14)	3.52 (1.85–6.70) ^*∗*^	0.001^*∗*^
	No	571	33	1.0	1.0	
Meat intake per week	No	227	15	1.0	1.0	
	1-2 days	285	30	1.59 (0.83–3.03)	0.73 (0.32–1.64)	0.451
	≥3 days	157	22	2.12 (1.06–4.21)	0.64 (0.25–1.63)	0.358
Fat intake per week	No	310	15	1.0	1.0	
	1-2 days	201	13	1.33 (0.62–2.86)	1.40 (0.58–3.39)	0.445
	≥3 days	158	39	5.10 (2.73–9.53)	3.50 (1.60–7.65) ^*∗*^	0.002^*∗*^
Vegetable intake/week	<3 days	391	50	2.09 (1.18–3.70)	2.51 (1.23–5.04) ^*∗*^	0.010^*∗*^
	≥3 days	278	17	1.0	1.0	
Walk/ride bicycle	Yes	564	46	1.0	1.0	0.124
	No	105	21	2.45 (1.40–4.27)	1.83 (0.84–3.98)	
Vigorous sports	Yes	431	21	1.0	1.0	0.006^*∗*^
	No	238	46	3.96 (2.31–6.80)	2.70 (1.33–5.49) ^*∗*^	
Moderate sports	Yes	444	25	1.0	1.0	0.301
	No	225	42	3.31 (1.97–5.57)	1.44 (0.72–2.89)	
Means of transport	On foot	443	30	1.0	1.0	
	By vehicle	226	37	2.42 (1.45–4.01)	1.56 (0.76–3.20)	0.224
Time spent by watching TV	<3 hrs	303	24	1.0	1.0	
	3–5 hrs	248	24	1.22 (0.67–2.20)	0.95 (0.47–1.91)	0.898
	≥5 hrs	106	17	2.02 (1.04–3.91)	1.43 (0.62–3.31)	0.410

^*∗*^Statistically significant at *p* value <0.05

## Data Availability

All data generated or analyzed during this study are included within this article.

## Abbreviations

AOR: Adjusted odds ratio
BAZ: BMI for age *Z*-score
BMI: Body mass index
CDC: Center of Disease Control
DBM: Double burden of malnutrition
FANT: Food and Nutrition Technical Assistance
GPAQ: Global Physical Activity Questionnaire
HDDS: Household Dietary Diversity Score
NCD: Noncommunicable diseases
SES: Socioeconomic status
SPSS: Statistical Product and Service Solution
TSFT: Triceps Skin Fold Thickness
WHO: World Health Organization.
